# TraceLAB: A MATLAB Toolbox for Interindividual Synchrony Analysis of Facial Expression and Head Movement Data Acquired via Trace

**DOI:** 10.3390/e28050503

**Published:** 2026-04-29

**Authors:** Felix Carter, Mike Richardson, Danaë Stanton Fraser, Iain D. Gilchrist

**Affiliations:** 1 School of Psychological Science, University of Bristol, 12a Priory Road, Bristol BS8 1TU, UK; i.d.gilchrist@bristol.ac.uk; 2 Department of Psychology, University of Bath, 10 West, Claverton Down, Bath BA2 7AY, UK; mr945@bath.ac.uk (M.R.); pssds@bath.ac.uk (D.S.F.)

**Keywords:** facial expression analysis, interindividual synchrony, shared information processing, correlated component analysis, surrogate synchrony, intersubject correlation, affective computing, mutual information

## Abstract

Facial expressions transmit information about internal states, both during social interaction and in response to shared stimuli such as films. When individuals view the same content, synchrony in their expressions reflects shared information processing, and the degree to which their expressions correlate indicates how similarly their perceptual and affective systems are responding to the common input. This makes interindividual expression synchrony a potential marker of engagement and subjective experience. However, the acquisition and analysis of facial data pose both ethical and technical challenges to researchers. ‘Trace’ is a research media player implemented in PsychoPy’s online platform Pavlovia, which captures anonymised facial landmark coordinates through a webcam, without the ethical and technical constraints of capturing and storing video images of participants. Nonetheless, its usefulness is currently limited due to the lack of available preprocessing and analysis tools. This paper describes the functionality of TraceLAB, a MATLAB-based toolbox designed for the preprocessing of Trace data: specifically, the formatting, aligning, and filtering of data. In addition, TraceLAB implements some novel analysis techniques to allow researchers to quantify interindividual synchrony of expressions (through correlated component analysis) and head movements (through Surrogate Synchrony), which may be interpreted as measures of shared information processing. These techniques are demonstrated here on both simulated and real datasets.

## 1. Introduction

### 1.1. Background

Emotional expressions are fundamental, non-verbal channels of communication [1] that shape the dynamics of interpersonal relationships [2]. Specific emotions tend to be associated with movements of similar configurations of facial muscles across cultures [3]. The strength of these muscle contractions may indicate the strength of the emotion [4], while variations in the expression of a given emotion may indicate whether the emotion is controlled, spontaneous, simulated, etc. [1]. It should be noted that this ‘basic emotion’ perspective (which treats emotions as discrete states which are universally observed) has been challenged by constructionist perspectives that emphasise the importance of context and conceptual knowledge in emotion perception (e.g., [5,6]).

Irrespective of whether facial expressions map onto discrete emotion categories, they remain a rich source of information about an individual’s affective state. Furthermore, the co-occurrence of similar expressions between interacting individuals (i.e., interindividual synchrony of expressions), particularly smiling, appears to be a fundamental mechanism of coordinating interaction dynamics; smiling mimicry occurs rapidly (within ~200 ms; [7]), correlates with team cohesion on a coordination task, and acts as a signal to change the collective strategy [8]. Furthermore, smiling synchrony encodes information about both the emotional [7] and social context of the interaction [9]. Affective interindividual synchrony more broadly appears to serve efficient information exchange, interpersonal emotion regulation (nonverbal synchrony between therapists and clients appears related to therapy outcomes [10]) and social bonding [11,12].

Yet despite a primarily communicative function, emotional expressions frequently occur in the absence of others [1], and the intensity of expressions reflects emotional responses to stimuli even in situations where there is little opportunity or incentive to communicate, such as when viewing films alone [4] or as part of an audience of a musical performance [13]. Furthermore, synchrony in emotional responses (i.e., both autonomic physiological and self-reported) during film viewing has been observed for merely co-present (i.e., non-interacting) dyads during film viewing [14], while the strength of synchrony predicts ratings of social connectedness during activities that do not involve direct interaction [15]. Zygomatic synchrony (i.e., of facial muscles) predicts affiliative ratings following co-present film viewing for previously unacquainted dyads and appears significantly related to physiological synchrony during the viewing of positively valenced (but not negatively valenced) films [16]. Another study [17] found that friends who viewed amusing video clips together showed significant convergence in the expression of amusement, and this did not significantly decrease when they were separated by a partition, suggesting that synchronised expression occurs in response to films even in the absence of direct visual contact with other audience members.

This finding is particularly interesting from an information-theoretic [18] perspective. Because the partition eliminated visual feedback between viewers, the observed synchrony cannot be attributed to mutual influence. It must instead arise from the shared processing of the common stimulus. Each viewer’s face can be conceptualised as an output channel that encodes information about their ongoing perceptual and affective processing of a film; when viewers are engaged and processing the stimulus similarly (e.g., attending to the same events, experiencing similar emotional responses), their facial output channels should exhibit shared variance. Synchrony, in this framework, is an index of how similarly their individual systems are transforming common stimulus information into expressive behaviour. The mutual information between any two viewers’ facial time series quantifies the extent to which knowing one person’s expressions reduces uncertainty about the other’s, not because they are influencing each other but because both are being influenced by the same external source. As a quantification of shared information processing, expression synchrony thus offers a continuous measure of attentional and affective engagement that can be estimated for individuals or groups, making it a potentially powerful tool for studying affective processes at both levels.

While mutual information provides the conceptual motivation for this framework, TraceLAB implements linear correlation-based methods (Fisher-transformed cross-correlation coefficients for SUSY and generalised eigenvalue decomposition for CorrCA). Correlation captures shared temporal dynamics in the signals (i.e., if, while viewing the same stimulus, two viewers’ expressions change together, or they consistently exhibit similar movement patterns, this indicates shared information processing). Under Gaussian assumptions, correlation and mutual information are monotonically related, and correlation-based methods are widely used in synchrony research for their computational simplicity and interpretability. Nonlinear extensions remain a direction for future work (see Section 4).

It has been demonstrated that emotional responses to stimuli (e.g., films) are reflected in expressions [4], and that interindividual synchrony broadly (e.g., in physiology [19,20,21], neural activity [22,23] or bodily movement [24]) is positively related to audience engagement. While there is some evidence that the intersubject correlation (ISC) of emotion classification scores (derived from *FaceReader* [25]) correlates with self-reported audience experience, particularly for happiness [26] and sadness [13], and that intersubject correlation (ISC) and magnitude of emotion classification scores are seemingly independent [26,27], there is overall relatively little research that has examined expression synchrony in audiences. This may be due in part to methodological challenges associated with both the acquisition of suitable data and the lack of accessible tools for the preprocessing and analysis of expression synchrony. Challenges with the acquisition of expression data include the requirement for either wearable monitoring devices (e.g., facial electromyography; [9]) or for a less invasive method—high-resolution cameras combined with ideal experimental conditions (e.g., adequate lighting) that may be difficult to achieve in ecologically valid audience scenarios (such as darkened auditoriums or cinemas). Additionally, the acquisition and retention of video data may present additional challenges, both ethical (as participants are identifiable) and technical (as large video files must be retained, preprocessed, analysed, etc.).

Facial-landmark-based methods, whereby positional coordinates rather than video images are captured, help overcome these challenges. A tool for facial landmark data acquisition, *Trace* [28], was recently added to PsychoPy’s [29] online experimental platform, Pavlovia [30], representing an accessible, low-cost and convenient method for capturing facial landmark data that overcomes many of the issues typically associated with the acquisition of expression data; as only facial landmark coordinates are retained, data is anonymous at the point of capture and file sizes are far smaller than for video data. Furthermore, *Trace* simply requires a computer webcam, and as it is implemented in Pavlovia, can be deployed remotely. Nonetheless, the preprocessing and analysis of facial landmark data remains a challenge due to the large number of data points that are typically analysed (e.g., 68 from face-api.js, *OpenFace*, etc.). These challenges are exacerbated for individual synchrony analysis, which requires data to be comparable in terms of length, sampling rates, etc.—features not typically present in *Trace* data (see below).

TraceLAB is a user-friendly MATLAB [31] toolbox that can be used for the preprocessing of Trace data and additionally offers approaches novel in the context of facial landmark analysis to quantify synchronous movement between individuals. These are described in the following sections.

### 1.2. Overview of Trace

Trace [28] is a research media player originally developed for measuring attentional engagement by recording anonymised facial landmark data via webcam. It uses face-api.js [32], a TensorFlow.js-based library, to capture X/Y coordinates for 68 facial landmarks and emotion classification scores for seven basic discrete emotions (happiness, sadness, fear, anger, disgust, surprise, and neutral) using a convolutional neural network with depthwise separable convolutions.

Due to its relatively high spatial (i.e., 68 landmarks) and temporal dimensionality (i.e., a mean sampling rate of ~5 Hz), Trace provides a relatively large amount of facial landmark data, which, combined with its availability and accessibility (it is implemented in Pavlovia, PsychoPy’s free-to-use online platform; [30]) makes it a potentially powerful research tool, especially for the large-scale (e.g., online) collection of meaningful naturalistic behaviour during video watching. The utility of head movement (quantified through nose tip movement, as recorded by *Trace*) as a measure of attentional engagement during video watching has already been demonstrated in previous studies [27,33]. TraceLAB implements the approach taken in those studies to operationalise head movement and thus may be useful for researchers looking for an accessible, continuous and objective measure of attentional engagement in video content.

Despite the clear potential utility of *Trace* as a research tool, there are currently some barriers to entry—in particular, the lack of available tools for the preprocessing (i.e., cleaning and formatting) of Trace data. Preprocessing is particularly important given that *Trace* does not capture data at a consistent sampling rate, and is therefore prone to missing or misaligned data. TraceLAB implements a user-configurable preprocessing function that automates the cleaning, filtering, processing and alignment of data as described below.

The alignment of data is particularly important when analysing interindividual synchrony, which typically requires recordings matched in length and sampling rate. Interindividual synchrony of facial expression may be a particularly powerful measure of subjective state (i.e., affective, attentional), but there are currently no validated methods for quantifying the intersubject correlation (ISC) of facial expressions. TraceLAB implements one such approach—correlated component analysis—which is often used to quantify the ISC of neural activity [22] captured though electroencephalography (EEG) recordings, which have a similar high spatiotemporal dimensionality as the facial landmark data captured by Trace. TraceLAB also allows users to quantify the ISC of head movement through the application of the Surrogate Synchrony (SUSY) procedure [34] to head movement data. This may be a useful measure of engagement given that head movement amplitude in general indexes engagement in the online viewing of video content [33], and the interindividual synchrony of bodily movement correlates with engagement in a live concert [24]. However, the ISC of head movement specifically has not, as far as we are aware, been validated as an index of engagement.

The following sections briefly describe the main functions of TraceLAB, although users are encouraged to consult the documentation and source code [35] that describe configurable input parameters for each function. Demonstrations of these functions as applied to simulated and real data are included in the following sections.

## 2. Methods

TraceLAB was developed in MATLAB [31] v2024a. No additional toolboxes are required for its use. For more information, please see the official TraceLAB GitHub page [35].

### 2.1. Overview of Preprocessing in TraceLAB

TraceLAB’s preprocessing function (*t_preproc*) is required to be the first function applied to Trace’s output data, as all TraceLAB analysis functions assume a particular data structure. It requires as its input argument the file path to the folder that contains the user’s Trace data (i.e., .csv files as downloaded from Pavlovia following data collection). Typically, users will also have to specify column names that contain the *participant* ID and any grouping variables by which they wish to separate their data (e.g., if their study contains different experimental conditions, or features different stimuli shown to the participants, these can be specified and TraceLAB will separate data according to these grouping factors).

**Figure 1:**
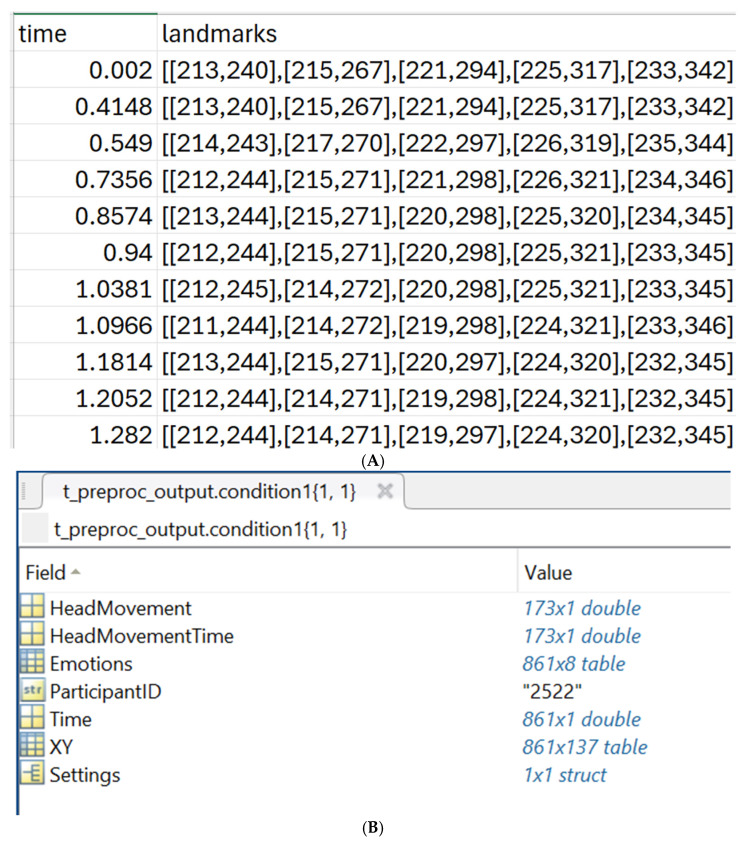
(**A**) Raw output from Trace showing landmark data formatted as JSON paired vectors, and inconsistent sampling. (**B**) Output from *t_preproc* function as it appears in MATLAB.

As Trace data is captured through an online platform, it can often produce large amounts of missing data, and the sampling rate can be inconsistent [33] (see also Figure 1A). *t_preproc* filters data by allowing users to set a maximum threshold of missing data within a recording before it is excluded from the dataset, and performs alignment (through the interpolation of each landmark’s X/Y coordinates using MATLAB’s *interp1* function, and trimming according to various user-defined parameters; see [35] for additional details).

Figure 1B shows the output structure of *t_preproc* as it appears in MATLAB. It contains a table of the XY coordinates of the 68 landmarks stacked into 136 columns, which are ordered *x*1, *y*1, *x*2, *y*2…*x*68, *y*68, with an additional *time* column on the left. The *Settings* field contains the input parameters of the preprocessing function (for bookkeeping and for subsequent functions to access).

The *HeadMovement* column contains head movement data quantified using the approach in [33], in which the magnitude of head movement was shown to significantly negatively correlate with self-reported engagement (i.e., more engaged participants fidgeted less). Second-to-second nose energy (i.e., movement of the tip of the nose; landmark #34) is used for this measure, as the nose tip is stationary relative to the rest of the face. The inter-ocular distance (IOD) of the eyes is taken from the landmark positions, and then scaled from pixels to centimetres with a scaling factor of 6.3/IOD-in-pixels (6.3 cm is the average IOD of an adult; [36]).

### 2.2. Head Movement Synchrony

**Figure 2:**
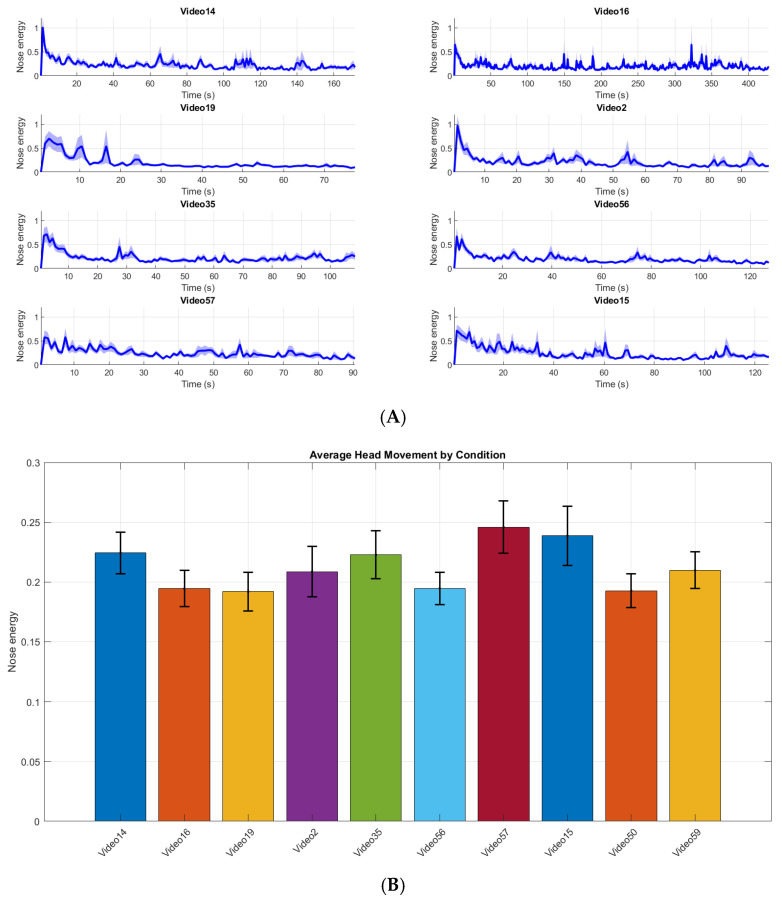
(**A**) Output from *t_plot_movement* showing mean head movement by condition in nose energy per second (shaded region shows standard error). (**B**) Output from *t_plot_movement* where data were aggregated over the entire recording in the prior *t_movement_summary* step (error bars show standard error). This data is from [37].

TraceLAB contains many functions for analysing and visualising head movement. For users simply interested in the magnitude of head movement, these data can be simply calculated and visualised using *t_movement_summary*, at many levels of aggregation (e.g., participant, condition, over time or averaged across the entire recording—see Figure 2).

TraceLAB also implements an adaptation of the Surrogate Synchrony [34] (SUSY) procedure for calculating intersubject correlation (ISC) in single-channel time series data (in this case, head movement, although emotion classification scores may also be used within TraceLAB for this purpose, as in [37]).

SUSY performs cross-correlations up to a user-defined maximum lag value (*l*) specified relative to the sampling frequency of the given data (*f*). Data from one member of each dyad is shifted in both directions relative to the other (up to ±*l* samples), and the instantaneous correlation between the two (i.e., *l* = 0) is also calculated. Thus, the number of coefficients obtained by SUSY for each segment and dyad is 2 (*lf*) + 1. For the context in which TraceLAB was developed, where synchrony is driven by the shared information processing of a common external stimulus rather than mutual direct influence, the lag parameter can be used to account for individual differences in response latency. The cross-correlation structure reveals not just whether viewers are responding similarly, but whether their responses are temporally aligned with the stimulus events.

Cross-correlations undergo Fisher Z transformations, allowing cross-correlation values for each segment (across lags) to be aggregated to provide a general measure of synchrony for each dyad. SUSY also implements a circular shuffle procedure to produce a surrogate dataset which shares the same statistical properties of the original data (mean, standard deviation, etc.) but the temporal structure is destroyed. The cross-correlations obtained from this surrogate dataset are compared to the original to provide an effect size (*ES*; Equation (1)) comparable to Cohen’s *d*, which is given by

(1) $$ES=\frac{\mu_{real}-\mu_{surrogate}}{\sigma_{surrogate}}$$

As with head movement data, output from TraceLAB’s SUSY function (*t_susy*) can be aggregated across different levels using *t_susy_summarise*.

### 2.3. Emotion Classification Synchrony

Richardson et al. [37] found that for viewers of emotionally evocative film clips, the ISC (quantified by SUSY), but not magnitude, of emotional classification scores significantly differed for the expected emotion (i.e., that which the film was intended to elicit) compared to other emotions, suggesting that SUSY may provide a more sensitive measure of affective response than emotion classification scores alone. As well as the extraction of average classification scores per emotion, TraceLAB offers an analysis of interindividual synchrony in classification scores via SUSY. As SUSY requires single-channel data, t_susy requires that the user must specify a particular column of data (e.g., *angryDetection*).

### 2.4. Correlated Component Analysis

Correlated component analysis (CorrCA) identifies dimensions of multivariate data that maximise shared information across participants [38]. Originally developed for quantifying the interindividual synchrony of neural activity [21,39], it solves for linear combinations of signals (in this case, facial landmark movements) that are most correlated between individuals. In the context for which TraceLAB was developed, CorrCA effectively isolates the components of expressive behaviour that reflect the common processing of a shared stimulus. CorrCA solves the generalised eigenvalue problem (Equation (2)):

(2) $$R_{B}W=\Lambda R_{W}W\;$$

where $R_{W}$ and $R_{B}$ are the within- and between-subject covariance matrices across N participants. The eigenvectors W define synchronised components, and *Λ* is a diagonal matrix whose elements $\lambda_{k}$ are the eigenvalues that quantify the synchronisation strength for each component. These are normalised to yield interpretable ISC values (i.e., the $N^{-1}$ term ensures the absolute value of ρ_k≤1 and is interpretable as a correlation-like measure; Equation (3)).

(3) $$\rho_{k}=\frac{1}{N-1}\lambda_{k}\;$$

For each component k, $\rho_{k}$ represents the overall intersubject correlation. Per-subject contributions are computed (Equation (4)) as

(4) $${ISC}_{i}=\frac{diag\left(W^{T}R_{B,i}W\right)}{diag\left(W^{T}R_{W}W\right)}$$

where *W^T^* are the transposed eigenvectors *W*. *R_bi_* and *R_wi_* are, respectively, the individual between- and within-subject covariance matrices.

To characterise moment-to-moment fluctuations in interindividual synchrony, a sliding window is applied (default length = 3 s). Rather than recomputing eigenvectors for each window (which would yield non-comparable components across time), the global projection matrix *W* (derived from the generalised eigenvalue decomposition of the full-dataset covariance matrices) is applied to window-specific covariance estimates. For each time window centred at *t*, the ISC for component *k* is computed (Equation (5)) as

(5) $${ISC}_{k}\left(t\right)=\frac{W_{k}^{T}R_{b}^{\left(t\right)}W_{k}}{W_{k}^{T}R_{w}^{\left(t\right)}W_{k}}$$

where $R_{w}^{\left(t\right)}$ and $R_{b}^{\left(t\right)}$ are the within- and between-subject covariance matrices estimated from the windowed data. This produces a continuous ISC-per-second trace that reveals the temporal dynamics of synchrony while maintaining component identity across the recording.

To interpret synchronised components in the original feature space, TraceLAB computes the forward model (Equation (6)):

(6) $$A=R_{w}W{\left(W^{T}R_{w}W\right)}^{-1}$$

where $R_{w}$ is the full-dataset within-subject covariance matrix. The forward model maps the synchronised components back into the original feature space; the columns of *A* represent spatial loading patterns, where large absolute values indicate facial landmarks that contribute strongly to a given synchronised component. Multiplying this forward model with the projected component time series reconstructs the original feature contributions (Equation (7)):

(7) $$\hat{X}=A\cdot \left(W^{T}X\right)\;$$

The columns of *A* represent spatial loading patterns, where large absolute values indicate features (i.e., facial landmarks) that contribute substantially to a given synchronised component. A regularisation (shrinkage) parameter (*γ*) is applied to stabilise covariance estimates, reducing sensitivity to random noise (e.g., blinking). TraceLAB sets γ=0.1 as the default. To evaluate the robustness of results to this choice, a gamma sensitivity analysis is performed across γ=[0.05, 0.1, 0.2, 0.5, 1.0] (see Section 3.4).

### 2.5. Data Preparation

It is worth noting that TraceLAB’s CorrCA function (*t_corrca*) does not take as input the full X/Y table of facial landmarks. This is for mathematical and theoretical purposes; CorrCA requires scalar time series as inputs (i.e., not vector pairs), and treating X/Y coordinates as separate features would yield components that isolate horizontal from vertical movement, which is often anatomically implausible and, in any case, would result in components that are difficult to interpret. Therefore, each landmark’s trajectory is compressed into a single time series. By default, TraceLAB uses the vector norm (Equation (8)), which takes the distance from the landmark’s mean-centred position:

(8) $$r_{t}=\sqrt{x^{2}+y^{2}}\;$$

Users may also select motion amplitude (Equation (9)), which takes the distance from the previous frame’s coordinates:

(9) $$d_{t}=\sqrt{{\left(x_{t}-\;x_{t-1}\right)}^{2}+{\left(y_{t}-\;y_{t-1}\right)}^{2}}\;$$

## 3. Results

The analyses presented below are intended to demonstrate TraceLAB’s functionality and illustrate its outputs rather than to validate its measures as indices of engagement or affective response. Formal validation against independent criteria remains an important direction for future work.

### 3.1. SUSY—Example with Simulated Data

To demonstrate the efficacy of SUSY on head movement data, a simulation of head movement signals was performed for 30 participants during a 2-min recording (sampled at 1 Hz). A synchronous signal was injected for all participants between 70 and 80 s to test dyadic synchrony detection. Individual head movement was simulated as white Gaussian noise. A synchronous template was created as a 0.5 Hz sine wave without tapering. Synchrony strength was controlled by a mixing parameter (α = 0.7): *x_s_y_n_c* = α·*template* + (1 − α)·noise within the synchronised window only.

**Figure 3:**
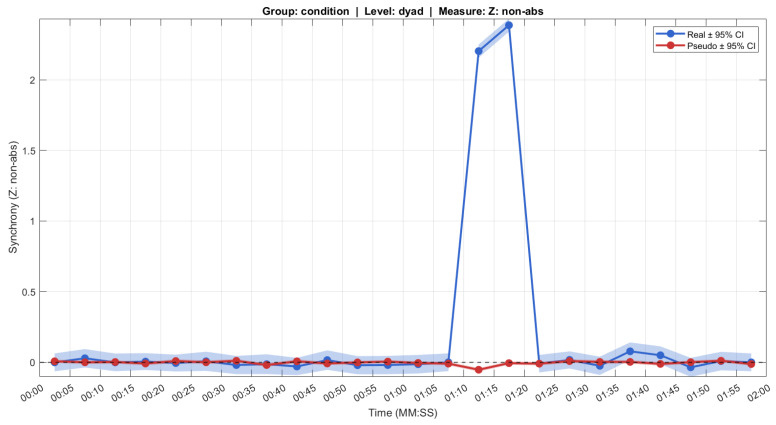
Output by *t_plot_susy* showing mean head movement synchrony (Fisher Z-transformed cross-correlation coefficients) for a simulated noisy signal with strongly synchronous signal injected at 70–80 s (blue), and the pseudo cross-correlation coefficients from surrogate data (red). Shaded regions show 95% confidence intervals.

The simulation included 5 s segments for time-resolved analysis, with the synchrony event spanning segments of 70–80 s. No systematic individual differences were added to ensure that any detected synchrony would reflect the injected signal. The SUSY analysis used a *maxlag* of 0 to focus on instantaneous correlation. The results of *t_plot_susy* performed on this simulated dataset are shown in Figure 3.

To test whether the injected synchrony exceeded surrogate baselines, we compared the Fisher-transformed cross-correlations (*Z*) during the synchrony window (70–80 s) to those obtained from circularly shuffled surrogate data (*Z_pseudo*), pooling across all lags. The mean *real Z* (1.581) was significantly higher than the mean *surrogate Z* (−0.020; paired *t*-test: t (1304) = 44.60, *p* < 0.001). This confirms that SUSY reliably detects induced head movement synchrony above chance levels.

### 3.2. CorrCA—Example with Simulated Data

**Figure 4:**
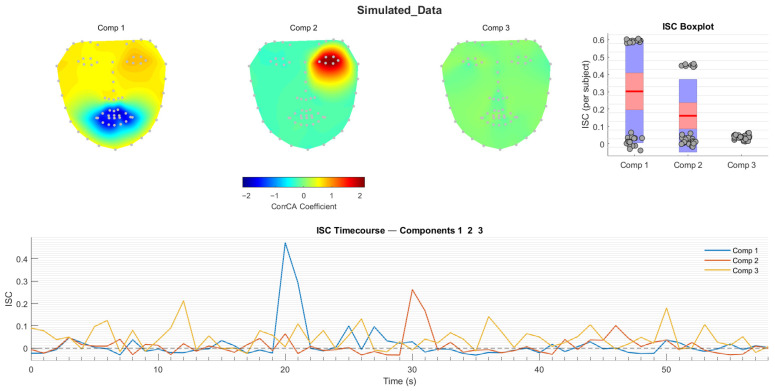
Output of *t_multiplot_corrca* on simulated data, with strongly synchronous signals injected for a subset of participants into the mouth region (20–21 s) and the left eye region (30–31 s). CorrCA conducted with γ = 0.1. Results are shown for the three components with the highest ISC values (i.e., those which capture the largest amount of shared variance). Top left: Topological facial plots showing the relative contribution of each landmark to each component. Note that negative loadings in the mouth component reflect eigenvector sign ambiguity and are not interpretable as directional differences. Top right: Boxplots showing the ISC per subject, where each box shows median and interquartile range. Bottom: ISC per second. Note the peaks in components 1 and 2 during the periods of injected synchrony.

Following *t_corrca*, the temporal and spatial characteristics, as well as the per-participant ISC values, can be visualised using *t_singleplot_corrca* (or all three can be visualised together using *t_multiplot_corrca*). For Figure 4, which demonstrates output from *t_multiplot_corrca*, facial movement was simulated for 30 participants during a 1 min recording (sampled at 5 Hz), with strongly synchronous signals injected into specific regions during specific time windows for subsets of participants. Two independent synchrony events were created: mouth synchrony for participants 1–15 during the interval from 20 to 22 s, and left eye synchrony for participants 16–25 during the interval from 30 to 32 s.

Beginning with TraceLAB’s standard 68-landmark face template, between-subject variance was simulated by adding independent Gaussian noise to each landmark’s position for each participant (σ = 5.0). Temporal dynamics were simulated through independent white noise added at each timepoint.

Each facial region was assigned a unique oscillatory signal (mouth: sin(*t*) + 0.5sin(2*t*); left eye: cos(2*t*) + 0.2cos(4*t*); right eye: sin(1.5*t*) + 0.3sin(3*t*); jawline: 0.5 + 0.5sin(0.8*t*); nose: sin(3*t*) + 0.2sin(6*t*).

Synchronous signals were injected only during specified windows for designated participant subsets. Phase jitter was applied by circularly shifting each participant’s signal by −2 to +2 frames (±0.4 s) and amplitude was scaled between 0.8 and 1.2 to provide movement magnitude variation.

As shown in Figure 4, CorrCA correctly identifies the two distinct shared movement components. Note that the negative direction of the mouth’s landmark contribution to Component 1 is a consequence of eigenvector sign ambiguity (*Cv* = *λv* holds for ±*v*), which is resolved arbitrarily by the solver. Interpretation of CorrCA should thus focus on the relative pattern of loadings, not their sign.

### 3.3. CorrCA—Example with Real Data

Trace and TraceLAB were applied to the Ryerson Audio-Visual Database of Emotional Speech and Song [40] (RAVDESS), a dataset of short videos (~3–4 s) of actors performing overt expressions of discrete emotions while vocalising. For this exercise, the discrete emotion was taken as the ‘condition’ label (i.e., *t_corrca* was performed on all ‘happy’ videos together, all ‘angry’ videos together, etc., with γ = 0.1).

**Figure 5:**
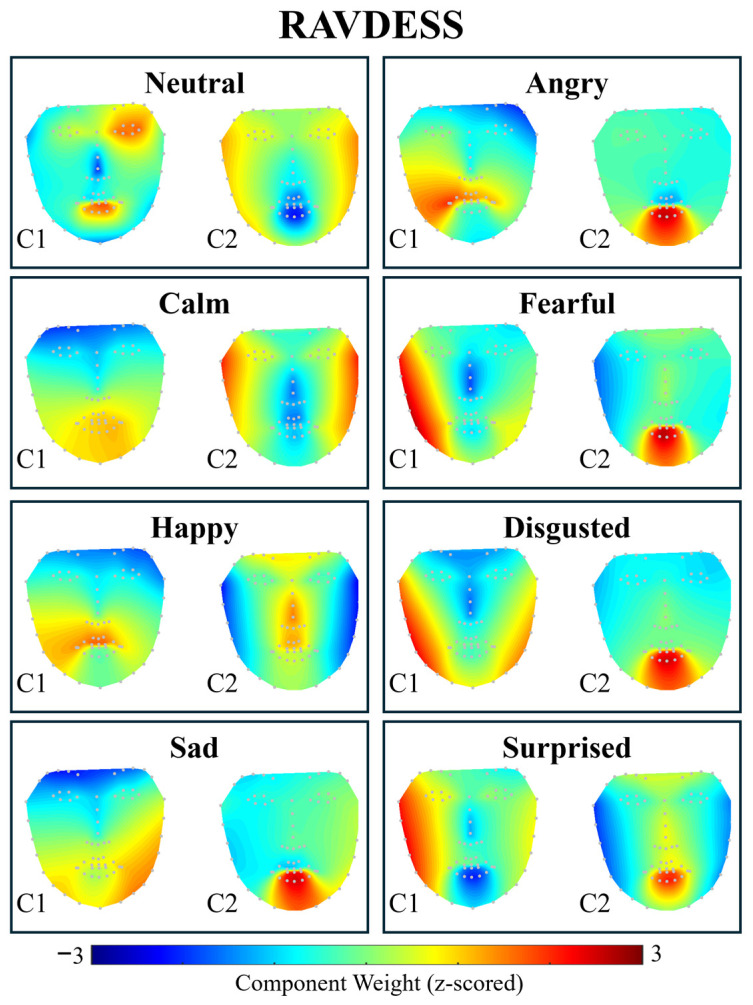
Output of *t_corrca* conducted on RAVDESS dataset with default parameters. Topoplots show the forward model weights for the first two CorrCA components. Warmer colours indicate facial landmarks with greater contribution to the component’s shared activity across participants. C1 captures the spatial pattern of facial movement with the highest intersubject correlation, with C2 representing the next most shared orthogonal pattern.

As shown in Figure 5, CorrCA identified movement in the mouth as the second largest shared movement component for the majority of conditions, likely due to the mouth movements made by actors during vocalisations. The ISC values per participant for each condition are shown in Table 1. Due to data length being insufficient to yield meaningful estimates of ISC per second, those are not reported.

**Figure 6:**
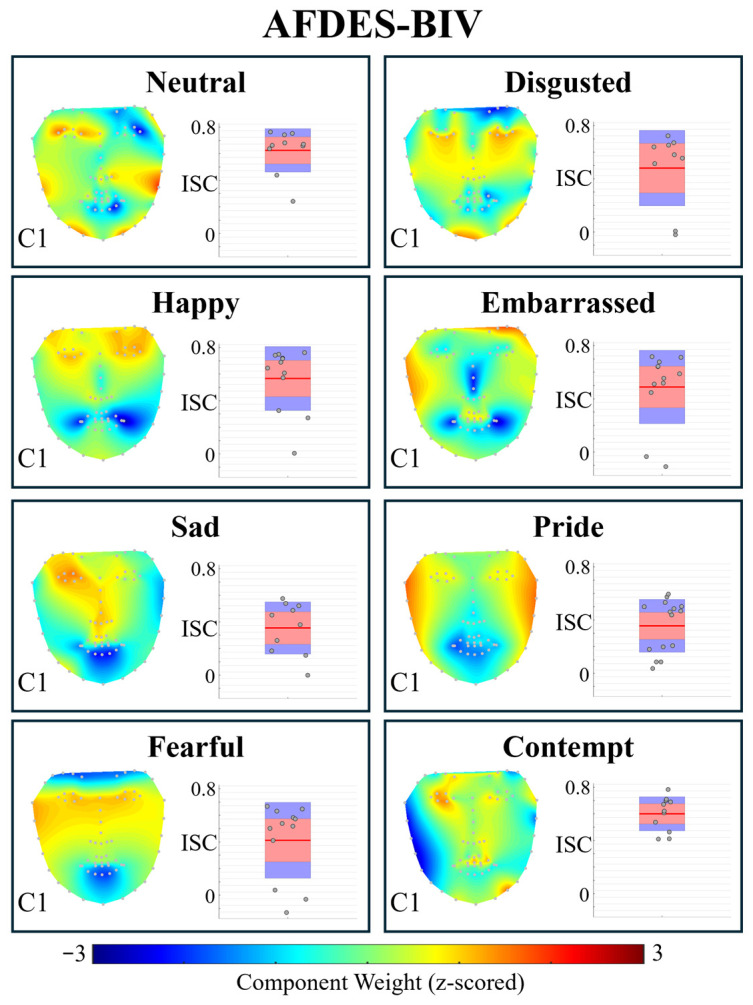
Output of *t_corrca* conducted on ADFES-BIV dataset with default parameters. Topoplot shows the forward model weights for the largest CorrCA component (i.e., that with the highest ISC). Warmer colours indicate facial landmarks with greater contribution to the component’s shared activity across participants. Boxplots show the distribution of per-subject ISC values for the same component; each point represents an individual participant’s ISC, indicating how strongly their component time course correlates with the group average. Boxes show the median and interquartile range.

Trace and TraceLAB were also applied to the ADFES-BIV [41] (Amsterdam Dynamic Facial Expression Set–Bath Intensity Variations) dataset, which features short (~1 s) videos of actors performing overt emotional expressions, but with no vocalisations. As shown in Figure 6, the largest component for each condition does not appear to be particularly spatially well-defined (possibly due to the relatively small amount of data which survived *t_preproc*—see Table 2), and the short length of the recordings which required using a smaller sliding window for CorrCA resulting in noisier covariance estimates (as they are based on fewer data points). As each dataset contains ~1 s of data, ISC per second estimates are not reported.

To test whether ISC differed across emotion conditions, we performed one-way ANOVAs on Component 1 ISC values. For the RAVDESS dataset, ISC varied significantly across emotions (*F*(7,1426) = 11.80, *p* < 0.001). For the ADFES-BIV dataset, ISC also varied significantly across emotions (*F*(7,83) = 2.83, *p* = 0.011). These results confirm that CorrCA captures differences in expressive consistency across emotions.

### 3.4. Gamma Sensitivity Analysis

**Figure 7:**
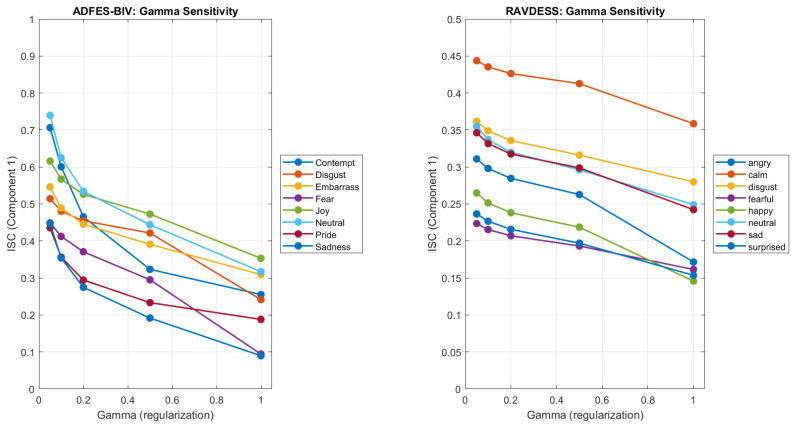
Component 1 ISC across regularisation parameters (γ = 0.05–1.0) for ADFES-BIV (left; *N* = 9–15 per condition) and RAVDESS (right; *N* = 95–192 per condition). Error bars show standard error. Rank-order of conditions is stable across γ for the larger RAVDESS dataset but varies for the smaller ADFES-BIV dataset, indicating that adequate sample sizes are required for reliable CorrCA estimates.

To assess whether the observed patterns depend on the choice of regularisation parameter, we repeated CorrCA across γ=0.05–1.0 for both datasets (Figure 7). In the larger RAVDESS dataset (N=95–192 per emotion), the rank-order of ISC values was highly stable across all γ. Calm consistently showed the highest ISC (range: 0.359–0.444), followed by Disgust (0.280–0.362), Neutral (0.249–0.355), and Sad (0.242–0.346). This stability indicates that the choice of γ has minimal impact on interpretation when sample sizes are sufficient.

In the smaller ADFES-BIV dataset (N=9–15 per emotion), the rank-order varied with γ. Neutral was highest at γ=0.05–0.2 (ISC range: 0.534–0.740), while Joy was highest at γ≥0.5 (ISC range: 0.353–0.473). This instability likely reflects the smaller sample sizes, which produce less stable covariance estimates. These results underscore the importance of adequate sample sizes for reliable CorrCA estimates, which is also reflected in the relatively poorer spatial definition of the components observed in the AFDES-BIV data (Figure 6) compared to those observed in the RAVDESS data (Figure 5).

## 4. Discussion

The paradigm for which TraceLAB was primarily designed (i.e., examining the synchrony of expressive response to a common stimulus) offers a particularly clear case for the information-theoretic analysis of facial expression. Unlike interaction paradigms, where synchrony may arise from multiple, conflated sources (e.g., mutual influence, shared context, common stimulus), synchrony in co-viewing has a single parsimonious explanation: shared processing of a common external information source. This simplifies interpretation considerably. Intersubject correlation in facial expressions can be directly interpreted as evidence that viewers’ perceptual–affective systems are extracting similar information from the stimulus at similar times. From this perspective, TraceLAB’s measures (CorrCA components and SUSY coefficients) quantify how much stimulus information is encoded across individuals.

A potential concern is whether the high ISC observed for neutral/calm expressions should be interpreted as “synchrony” in the same sense as the synchrony of expressive movements. We argue that it should. From an information-theoretic perspective, the mutual information between two viewers’ facial time series is high when both are still, and this reflects shared information about the stimulus (i.e., that it did not elicit a strong expressive response). The absence of movement is as informative as its presence; both convey information about how the viewer is processing the stimulus. This interpretation is supported by the pattern of results in the larger RAVDESS dataset, where Disgust (an expressive emotion) consistently outranks Neutral across all regularisation parameters (see Section 3.4). Thus, CorrCA captures both shared stillness and shared expressive movement, each reflecting meaningful aspects of the viewing experience [26,33].

It is nonetheless perhaps surprising that in the RAVDESS dataset, Calm expressions show consistently higher ISC values than highly stereotyped expressions (e.g., Surprise, Disgust). This pattern may reflect the low variance of calm expressions inflating the ISC estimates from CorrCA, or could indicate that the vocalisations accompanying calm speech produce more consistent mouth movements between actors than emotional expressions do. Distinguishing these possibilities requires further investigation.

### 4.1. Open Research Questions

In this paper, a high-level overview of TraceLAB’s functionality and simple demonstrations of its efficacy are presented. However, it is worth noting that this is a preliminary version of a toolbox that has much scope to grow and be adapted. It is entirely open source, and while it is intended to be of practical use to researchers ‘out of the box’, users are encouraged to tweak functions to their own purposes. Indeed, there are many methods of interindividual synchrony analysis that have been considered for, or are partially integrated into, TraceLAB and will be developed further. Dynamic time warping [42], for example, provides a measure of pairwise signal similarity that may prove to be useful in dyadic expression analysis. Likewise, wavelet analysis techniques have been used previously as a measure of the ISC of bodily movement [43,44]. Beyond linear correlation-based methods, mutual information and transfer entropy could capture nonlinear dependencies between viewers’ facial expressions, while wavelet-based phase synchrony offers a complementary approach to tracking time–frequency alignment. However, the utility of these methods in the context of expression analysis remains to be seen, and so have not been fully implemented in the current version of TraceLAB. While the utility of CorrCA for analysis of expression synchrony has also not been validated, it was deemed a potentially useful method for the purpose, especially in an exploratory capacity, as data can be projected through the component space across the dimension of time, individual (i.e., participant) or feature (i.e., landmark). Thus, it may be of use to researchers wishing to quantify ISC across any (or all) of these dimensions of their data.

While further research is needed to validate CorrCA of facial expression as a measure of affective or attentional state, framing synchrony as shared processing of stimulus information provides a basis for generating testable predictions. For example, manipulations that increase engagement (e.g., compelling narratives) should increase mutual information between viewers’ facial expressions, while distractions should decrease it. The relationship between synchrony and self-reported engagement can thus be understood as a validation check: does greater shared information processing correspond to what viewers report experiencing? While this has been demonstrated for a range of other physiological responses (e.g., heart rate [19,20,21] and neural activity [22,23]) and behavioural measures (e.g., bodily movement in response to music [24]) as well as categorical, qualitative descriptors of facial expressions [13,26], it has not yet been demonstrated via CorrCA of facial landmark movement or SUSY analysis of head movement. This framing also suggests a more specific prediction: the mutual information between viewers’ expressions should peak during moments of highest narrative or emotional intensity, when the stimulus is most information-rich and demands coordinated attentional and affective responses. Moment-to-moment fluctuations in the ISC of facial expression, as provided by *t_corrca*’s sliding window analyses, or of head movement, as provided by *t_susy*, offer a way to test this hypothesis directly.

### 4.2. Limitations

A distinct challenge for this approach regards the treatment (i.e., preprocessing) of facial landmark data for the purposes of expression synchrony analysis, as it is not currently clear which are the most appropriate mathematical operations for this purpose (although the answer will often depend on the research question posed). For example, with regard to the appropriate method for combining X/Y coordinates into a single vector series per landmark, the decision to use Euclidean distance displacement measures (i.e., vector norm and motion amplitude) was motivated by observations that such displacement measures appear effective for the expression similarity analysis of video data [45], and the results of testing on simulated data (presented here). However, alternative methods of preprocessing and analysing facial landmark coordinates (as well as methods for quantifying the ISC of expressions derived from landmark coordinates, of which CorrCA is just one example, albeit seemingly a particularly suitable one) ought to be explored. For example, a Laplacian filter may be a useful preprocessing tool, given its apparent utility in landmark detection [46,47], and as such is included in TraceLAB but not described here as there is currently no theoretical justification for its use in expression analysis. Region-based analyses may also be appropriate as a method of reducing the dimensionality of the 136-channel landmark data output by Trace.

A related consideration is the loss of directional information when compressing X/Y coordinates to scalar magnitudes. For example, two viewers who raise their eyebrows (upward movement) and two viewers who furrow their brows (downward movement) would produce similar magnitude time series, and CorrCA would treat both as similarly synchronised despite opposing directional responses. This may be more consequential for facial regions where direction conveys emotional meaning (e.g., eyebrows for surprise vs. anger) than for regions where movement magnitude alone is informative (e.g., mouth opening during smiling). Users for whom directional information is critical may wish to use the full 136-channel X/Y data in *t_corrca*, but should consider the potential difficulty of interpreting such output, especially as it would be incompatible with the facial topoplot function, which expects the forward model to have 68-rows i.e., one per landmark. Region-specific directional features may be a more suitable approach and will be explored for future implementations of TraceLAB.

Finally, it is worth noting that the use of landmark data, as opposed to video data, for expression analysis (both in general and for quantifying interindividual synchrony specifically) may be inherently limited due to the information loss that occurs when expressions are reduced to landmark coordinates alone, e.g., by discarding lighting and texture information that automated Facial Action Coding System [48] (FACS) algorithms rely on for Action Unit (AU) detection [49,50]. Initial attempts were made to implement a FACS detection function into TraceLAB; however, it was largely unsuccessful except for smiles, which appear to be detectable from landmark coordinates alone reasonably robustly. While this feature has not yet been formally added to TraceLAB, it likely will be in future, given evidence that smiling appears to be the facial action most commonly synchronised between interacting individuals [8] and seemingly most closely related to self-report experiences of theatrical [25] and musical performances [13].

The information loss that occurs when working with reduced features (i.e., landmark coordinates alone, relative to video images) is exacerbated by Trace’s inconsistent sampling rate and limited temporal resolution (~5 Hz), which constrains its ability to detect rapid facial movements, such as the ~200 ms smile mimicry observed in face-to-face interactions [7]. While this sampling rate is sufficient to capture slower expressive dynamics (e.g., emotional responses to narrative film [49]), it constrains the detection of synchrony at fine temporal scales, and thus, for applications requiring high temporal precision (e.g., turn-taking in conversation), Trace may not be a suitable platform. However, TraceLAB could be adapted for application to data captured through other platforms that may yield data with higher temporal resolutions (e.g., OpenFace). Additionally, future versions of Trace may benefit from increased sampling rates as webcam technology and browser-based tracking algorithms continue to improve. TraceLAB’s preprocessing functionality could also be expanded in future to accept data from other platforms that can process video images (e.g., OpenFace).

### 4.3. Conclusions

In summary, TraceLAB offers a standardised preprocessing pipeline for Trace facial landmark data, implements SUSY for single-channel synchrony analysis, and introduces CorrCA for multivariate expression synchrony, providing researchers with accessible tools to move from raw data to interpretable measures of shared information processing. Several limitations remain in the current implementation, including the loss of directional information when compressing X/Y coordinates, the lack of validation against independent measures of engagement, and the absence of empirically established default parameters (e.g., regularisation strength, sliding window length). There is considerable scope for further development, with clear opportunities including validation studies linking synchrony measures to independent indices of engagement, exploration of nonlinear methods such as mutual information and wavelet-based synchrony, and the integration of region-based analyses or, where feasible, action unit detection. Nonetheless, TraceLAB initiates a much-needed methodological exploration into quantifying interindividual synchrony from facial landmark coordinates, providing a foundation for future validation and theoretical advancement.

## Figures and Tables

**Table 1:** Mean ISC by condition for the RAVDESS dataset.

Condition	*N*	Mean ISC Per Subject (SE)
Neutral	95	0.32 (0.02)
Calm	192	0.41 (0.01)
Happy	189	0.20 (0.01)
Sad	192	0.27 (0.01)
Angry	192	0.25 (0.01)
Fearful	191	0.19 (0.01)
Disgusted	192	0.29 (0.01)
Surprised	191	0.20 (0.01)

**Table 2:** Mean ISC by condition for the ADFES-BIV dataset.

Condition	*N*	Mean ISC Per Subject (SE)
Neutral	10	0.63 (0.05)
Happy	12	0.57 (0.07)
Sad	10	0.35 (0.06)
Fearful	12	0.41 (0.08)
Disgusted	9	0.48 (0.09)
Embarrassed	12	0.49 (0.08)
Pride	15	0.36 (0.05)
Contempt	11	0.60 (0.04)

## Data Availability

Source code and all data reported can be found at https://github.com/FelixCarter/tracelab (accessed on 22 April 2026), except for the data presented in Figure 2, which can be found at https://doi.org/10.17605/OSF.IO/E8ZJ2.
